# Association of Circulating Levels of Inflammatory Cytokines and Chemotherapy-Associated Subjective Cognitive Impairment in a South African Cohort of Breast Cancer Patients

**DOI:** 10.3390/neurosci4040024

**Published:** 2023-11-07

**Authors:** Nicholas Keetile, Elzbieta Osuch, Antonio G. Lentoor, Tsakani Rasakanya

**Affiliations:** 1 Department of Pharmacology and Therapeutics, School of Medicine, Sefako Makgatho Health Sciences University, Ga-Rankuwa, Pretoria 0208, South Africa; mogomotsikeeti@gmail.com (N.K.);; 2 Department of Clinical Psychology, School of Medicine, Sefako Makgatho Health Sciences University, Ga-Rankuwa, Pretoria 0208, South Africa

**Keywords:** chemotherapy, breast cancer, cognitive impairment, chemobrain, neuroinflammation, cytokines, neuropsychology, self-perceived cognitive impairment, pharmacology

## Abstract

Background: The evidence links chemotherapy to cognitive impairment in breast cancer patients. This study assessed the link between subjective chemotherapy-related cognitive impairment and neuroinflammation in breast cancer patients. Methods: In a correlational study, 113 patients aged 21 to 60 years on chemotherapy regimens completed the Functional Assessment of Cancer Therapy-Cognition Test (FACT-Cog) as a measure of subjective cognitive functioning at three time points (baseline- T_0_, third cycle- T_1_, and sixth cycle- T_2_). The levels of inflammatory cytokines (interleukin-1 beta (IL-1β), interleukin-6 (IL-6), interleukin-8 (IL-8), and tumour necrosis factor-alpha (TNF-α)) were measured using an assay method and compared with the subjective cognitive impairment. Results: Midway through chemotherapy, higher levels of TNF-α were inversely linked with self-perceived cognitive performance, while higher levels of IL-1β were positively associated (*p* = 0.030). However, at the end of chemotherapy, only IL-8 (*p* = 0.50) was associated with higher self-perceived cognitive problems. Conclusions: The specific roles that various cytokines and their interactions may play in neuroinflammation or neuroprotection require further investigation.

## 1. Introduction

Global evidence indicates that breast cancer is the most commonly diagnosed female cancer, as reported by Christensen and Marck [1]. A persistent rise in new cases of this disease has been paralleled by improvements in survival rates since 1989, partly due to the availability of effective treatments according to Akram et al. [2]. Allemani et al. [3] reported that the 5 year survival for breast cancer in 17 countries globally increased to 85%. Chemotherapy is still regarded as the mainstay of treatment for breast cancer in many countries and regions of the world [4]. This treatment modality is typically administered in pulsed doses called cycles, and in the majority of cases, each cycle lasts practically 21 days [5]. The rationale behind chemotherapy cycles is to allow bone marrow to recover, since chemotherapy accounts for its suppression (referred to as myelosuppression). Additionally, adraimycin, methotrexate, fluorouracil, and cyclophosphamide are frequently utilised in combination (regimen) with one another as cytotoxic drugs during chemotherapy [6]. The main purpose of regimens is to increase cytotoxicity of tumour cells while preventing resistance to individual agents. In countries of low and middle income, standard doses of adjuvant chemotherapy regimens comprising of cyclophosphamide, methotrexate, fluorouracil (CMF) and fluorouracil, adriamycin, cyclophosphamide (FAC) have proved effective in combating a rise in cases of breast cancer over time [5,7]. These combinations of cytotoxic agents are implicated in a number of harmful effects experienced by patients.

Chemotherapy is implicated in the occurrence of subjective cognitive impairment in breast cancer patients [8,9]. According to Simó et al. [10] and Ono et al. [11], this phenomenon (referred to as “chemobrain”) manifests as impaired memory, attention, concentration, and executive function, and its symptoms are detected in up to 83% of patients during chemotherapy. Chemobrain symptoms persist for years in up to 35% of patients post-treatment and they can potentially impact the quality of life of patients [12,13]. Available data relating to chemobrain emanates from studies on Caucasian female breast cancer patients in Canada, Europe, and the United States of America [14], and this creates a knowledge gap as data on black African female breast cancer subjects is lacking. To date, the pathomechanism of chemobrain is plausible.

Chemotherapy-related neuroinflammation, which is characterised by elevated levels of inflammatory cytokines including interleukin-1 beta (IL-1β), interleukin-6 (IL-6), interleukin-8 (IL-8), and tumour necrosis factor-alpha (TNF-α) has been cited in the literature. It is propounded as a mechanism for the occurrence of chemobrain [15]. Moreover, perturbations in the levels of cytokine have been found to account for “sickness behaviour’, which is characterised by adaptive changes in cancer patients. The proposed mechanism behind this relationship is that cytokines can penetrate the blood brain–barrier into the brain. Such penetration is thought to occur at the circumventricular regions of the brain and it takes place through active transport [16]. Upon their entry into the brain, cytokines bind to endothelial receptors to consequently trigger the release of inflammatory mediators like nitric oxide, which induces brain damage and allows for more entry of inflammatory cytokines, and this ultimately results in cognitive impairment [17]. The current study sought to correlate chemobrain and circulating levels of these cytokines during chemotherapy in a group of female breast cancer patients in South Africa.

The significance of the current study is that it will sensitize health authorities locally and globally to strengthen their breast cancer care strategies and consequently improve the quality of life of patients.

## 2. Materials and Methods

### 2.1. Research Site, Research Design, and Participants

This study was conducted at an out-patient breast clinic of a recognised tertiary, academic hospital located in a semi-rural community of South Africa. This particular hospital was expressly selected on the basis that it is the only public health institution in the greater municipal area providing medical oncology services to poor black residents. The location of the research site is also accessible to the majority of cancer patients in the area and it adjoins the university laboratories, thus facilitating the handling of specimens immediately after collection from breast cancer patients.

A prospective, correlational, quantitative study was conducted. Complete data were obtained from all eligible participants who were recruited through purposive sampling techniques during the period of October 2018 to October 2010.

All breast cancer patients who were included in this study were chemotherapy-naïve and the majority of them had hormone-independent breast cancer type. A total of 127 female patients diagnosed with breast cancer stage II and stage III were enrolled in this study, of which 14 participants were excluded (7 had previous chemotherapy exposure, 1 had a current psychotic disorder, 6 were over the age limit of 60 years). The age range of participants was 21 to 60 and the final sample was comprised of 113 participants. 

### 2.2. Eligibility Criteria

Stages II and III breast cancer patients who met the inclusion criteria (e.g., not undergoing concurrent radiation therapy and having no concurrent psychotic disorder, epilepsy, and dementia) participated in the study. Patients who participated in this study were assigned to receive either cyclophosphamide, methotrexate, and fluorouracil (CMF) or fluorouracil, adriamycin, and cyclophosphamide (FAC) as per the following schedules: CMF (cyclophosphamide 100 mg/m^2^ orally, methotrexate 40 mg/m^2^ intravenously, 5 fluorouracil 600 mg/m^2^ intravenously every 3 weeks for 6 cycles) and FAC (fluorouracil 500 mg/m^2^ intravenously, adriamycin50 mg/m^2^ intravenously, cyclophosphamide 500 mg/m^2^ intravenously every 3 weeks for 6 cycles).

### 2.3. Data Collection

The treating oncologist and the nurse-in-charge assisted with the recruitment of participants. After verifying the participants eligibility, the nurse-in-charge outlined the aim of the study and gave details to them. Those meeting the inclusion criteria were asked to participate in the study and the researcher was introduced to those who consented. After verification, the researcher provided informed oral and written information about the study. It was highlighted to participants that participating in the study was completely voluntary and that it was permissible to withdraw from it at any time or refuse to participate. Participants were reassured that declined participation would not affect their continued care. All participants provided informed written consent.

### 2.4. Measures

#### 2.4.1. Sociodemographic and Health Information

Data collection involved completing a 5 min socio-demographic health questionnaire on age, gender, ethnicity, and health comorbidities at baseline.

#### 2.4.2. Subjective Cognitive Assessment

Each participant completed a 10 min self-reported cognitive measure, the Functional Assessment of Cancer Therapy-Cognitive Function (FACT-Cog) (version 3) at three points: at baseline–T_0_ (after cancer staging and prior to commencement of chemotherapy), third cycle-T_1_ (midway through chemotherapy), and sixth cycle-T_2_ (at the completion of chemotherapy). The FACT-Cog is a 37-item self-reported instrument that uses a 5-point Likert scale (0 = never to 4 = several times a day) to assess subjective cognitive function over a 7-day period.

#### 2.4.3. Blood Sample

Venous blood samples for circulating inflammatory markers were collected from participants by venipuncture into ethylene-diamine-tetra-aceticacid (EDTA) tubes by the nurse-in-charge. Immediately after collection, blood was centrifuged at 2000 revolutions per minute (rpm) for 10 min to separate the plasma. The plasma was stored in a deep freezer at −80 °C for further evaluation. An assay method was used to measure the levels of inflammatory cytokines. Blood samples were collected at three points: after cancer staging and prior to commencement of chemotherapy (baseline–T_0_), midway through chemotherapy (third cycle-T_1_), and at the completion of chemotherapy (sixth cycle-T_2_). The levels of cytokines were measured with the Multiplexing with Bio-Plex Pro human inflammation assays (Bio-Plex Pro™ human inflammation panel I) express assay, which is manufactured by Bio-Rad. The mode of operation of the assay was such that the captured antibodies, which are covalently coupled to beads, target cytokines of interest. The coupled beads competitively reacted with the antibodies–cytokines complexes. A sandwich complex was isolated through the addition of a biotinylated detection antibody following several washes to discard unbound proteins. Streptavidin-phycoerythrin conjugate, which is a fluorescence indicator, was added in the final analysis to form the detection complex.

### 2.5. Statistical Analysis

All statistical analyses were performed on Statistical Analysis System (SAS Institute Inc., Carey, NC, USA), Release 9.4 or higher, running under Microsoft Windows for a personal computer. Descriptive statistics (i.e., means, standard deviation, etc.) were performed on the participant’s health and demographic information. Baseline, during, and post-chemotherapy mean FACT-Cog scores and mean cytokine levels were compared by paired *t*-tests and the non-parametric Wilcoxon signed-rank test. Differences between baseline, during and post-chemotherapy cognitive scores, and cytokine levels were analysed. The percentages in each test group were compared by the Fisher exact test. All tests were two-tailed and held statistical significance at *p* < 0.05.

## 3. Results

### 3.1. Subjective Cognitive Function

All patients included in this study underwent all six cycles of chemotherapy assigned to them. Table 1 presents the descriptive statistics of the sample of participants in this study. Of a total of 113 patients, all of whom were black (100%), 77.4% in the CMF group and 80.0% in the FAC group showed significant subjective cognitive impairment (*p* < 0.05). In both groups, the majority of the participants (CMF = 29 (54.7%); FAC = 25 (41.7%) were in the age range between >50–60.

The results show that there was a significant decline in subjective cognitive function from baseline to the completion of chemotherapy. As it appears in Table 2, the mean scores of the CMF group on the FACT-Cog significantly decreased from 51.2 at T_0_ to 48.9 at T_1_ (*p* = 0.023). Furthermore, the CMF group shows an even larger subjective cognitive decline from 51.2 at T_0_ to 47.0 at T_2_ (*p* = 0.008). The mean change of 4.2 in subjective cognitive function scores from baseline to completion of chemotherapy means that there was progressive decline in subjective cognitive performance.

The results further Indicate that in the FAC group, the subjective cognitive performance as measured on the FACT-Cog decreased baseline to the end of chemotherapy. The mean FACT-Cog scores in this group declined significantly from 52.9 at T_0_ to 48.4 at T_1_ (*p* = 0.003). Similarly, there was a significant subjective cognitive decline from 52.9 at T_0_ to 44.0 at T_2_ (*p* < 0.001). The mean change in the FACT-Cog scores of the FAC group from baseline to chemotherapy cycle three is 8.9, and this shows a significant decrease in subjective cognitive functioning.

It is worth noting that the difference between the two mean scores (4.2 and 8.9) for groups CMF and FAC did differ significantly (*p* = 0.056) from baseline (T_0_) completion of chemotherapy (T_2_). The results of this study indicate progressive subjective cognitive decline in FAC treatment groups during courses of the chemotherapy treatment.

### 3.2. Inflammatory Markers over Study Period

Alterations in the measured levels of inflammatory cytokines in all 113 participants were recorded. In the CMF group, there were statistically non-significant alterations in IL-1β, IL-6, IL-8, and TNF-α at T_1_ (75.67 ± 108.27 pg/mL; 0.02 ± 0.03 pg/mL; 0.47 ± 2.06 pg/mL; 0.13 ± 0.23 pg/mL) when compared to T_0_ (86.68 ± 119.20 pg/mL; 0.02 ± 0.04 pg/mL; 1.11 ± 4.86 pg/mL; 0.12 ± 0.22), respectively. Similarly, there were statistically non-significant decreases in IL-1β, IL-6, IL-8, and TNF-α at T_2_ (76.92 ± 122.88 pg/mL; 0.01 ± 0.02 pg/mL; 0.58 ± 2.69 pg/mL; 0.14 ± 0.40) when compared to T_0_. In the FAC group, statistically non-significant increases were recorded in IL-1β, IL-6, IL-8, and TNF-α at T_1_ (3.38 ± 13.20 pg/mL; 13.29 ± 24.12 pg/mL; 272.29 ± 1162.44 pg/mL; 28.96 ± 38.44 pg/mL) when compared to T_0_ (1.72 ± 4.63 pg/mL; 13.85 ± 47.65 pg/mL; 113.55 ± 448.12 pg/mL; 36.54 ± 107.91 pg/mL), respectively. Similarly, there were statistically non-significant increases in IL-1β, IL-6, IL-8, and TNF-α at T_2_ (5.08 ± 15.32 pg/mL; 25.27 ± 66.40 pg/mL; 170.48 ± 524.21 pg/mL; 33.84 ± 36.18), respectively, when compared to T_0_.

### 3.3. Relationship between Inflammatory Markers and Subjective Cognitive Function

Pearson’s correlation was used to assess the relationship between inflammatory cytokine levels and subjective cognitive function (Table 3). At baseline, prior to starting chemotherapy, no relationship was found between cytokine levels and FACT-Cog scores. However, midway (cycle 3 of chemotherapy treatment) higher concentrations of IL-1β (*p* = 0.030) were associated with better self-perceived cognitive function, while for every 0.19 increase in unit of plasma, TNF-α was associated with an increase in self-perceived cognitive complaints (*p* = 0.042). Interestingly, after completion of chemotherapy (T_2_), only higher concentrations of IL-8 were found to be significantly associated with more self-perceived cognitive complaints (*r*(111) = 0.185, *p* = 0.050).

## 4. Discussion

This study found that, overall, breast cancer patients (stages II and III) assigned to CMF and FAC regimens had a decline in FACT-Cog scores from baseline to completion of chemotherapy. Similar findings emerged from previous studies which showed that breast cancer patients are susceptible to the cognitive decline following chemotherapy treatment [18,19]. Cheung and colleagues [17] showed that the administration of chemotherapy can lead to subjective cognitive decline. Furthermore, these results concur with those of Chen et al. [20], in which 42 patients with stage II and stage III breast cancer undergoing adjuvant chemotherapy (CMF and FAC) were evaluated, and they performed significantly poorly on attention, memory, and executive function tests (*p* < 0.05).

In this study, the FAC group performed slightly worse than the CMF group on the FACT-Cog test. Typically, anthracycline-based chemotherapy (adriamycin) exhibited increased susceptibility to cognitive impairment [21,22,23]. Likewise, Schagen and Wefel [24] found that chemotherapy-induced toxicity was associated with cognitive declines, based on both self-reported and objective cognitive tests.

While the findings of the current study concur with those of previous studies, the mechanisms for the occurrence of chemotherapy-associated cognitive impairment remains unclear. The subjective chemotherapy-related cognitive impairment, though varying in severity, was reported elsewhere in studies that explored this phenomenon in relation to the use of standard dose chemotherapy in cancer patients [25,26]. The present study’s result, which showed that FAC has been associated to more cognitive issues than CMF, would suggest that anthracyclines may be more neurotoxic than other chemotherapy medications [27]. Perhaps this observed correlation can be clarified by an experimental study design in the future. More multicentre and prospective cohorts with a large longitudinal follow-up design to explore the effects of anthracycline-based regimens are needed.

Alterations in the levels of inflammatory cytokines occurred during adjuvant chemotherapy in the current study. Congruent findings emerged from another study elsewhere in which increases in levels of selected inflammatory cytokines accompanied subsequent cycles of adjuvant chemotherapy [28]. In line with the findings of Wong et al. [29], adjuvant chemotherapy has been found to increase the levels of inflammatory cytokines. Our study found that out of four inflammatory cytokines, a negative correlation existed between the levels of TNF-α and mean FACT-Cog scores midway through chemotherapy treatment (T_1_). TNF-α has shown to play an important role in several important cognitive functions and central nervous system processes, such as regulation of the blood–brain barrier permeability [30]. Interestingly, elevated IL-1β concentrations at T_1_ were positively associated with self-perceived cognitive function. Studies have indicated that IL-1β may have a paradoxical effect in the central nervous system (CNS) [31]. On the one hand, it is linked to neurodegenerative processes. On the other hand, it may also signal neuroprotective properties in an injured CNS. However, this study sample was too small, and perhaps it would be important through further research to explore the possible neuroprotective role of IL-1β.

After the final dose of chemotherapy treatment, only IL-8 was found to be associated with more self-perceived cognitive complaints. This means that as the levels of inflammatory cytokines increased, cognitive performance decreased. Similarly, IL-8 plays an important role in higher order executive cognitive functions, learning, and memory [32]. It would be crucial to investigate the significance of chemotherapy-induced inflammation in a larger study. Importantly, in spite of the limitations of the study, the results do suggest that there may be an association between patients’ cognitive loss and anthracycline-based chemotherapy. Data on this particular correlation in the South African context is lacking, and this finding provides important information that can be incorporated in clinical settings of both oncology and neuropsychology but importantly provides justification for further research within the local context.

The fact that the majority of participants in both treatment groups in this study fell under the age range of 51–60 years could have influenced the results in a way, and this creates an important basis for future studies to explore this relationship in more detail.

This study was not without and limitations. Firstly, the small sample size could inhibit the generalisation of the findings. Secondly, only subjective cognitive function (with its potential to introduce bias) was assessed to the exclusion of the administration of a battery of objective neuropsychological testing. Thirdly, in this study, the homogeneity with respect to racial and socioeconomic profiles reflected by the study sample could have negative consequences in terms of generalizability of the results.

## 5. Conclusions

Chemotherapy may be associated with subjective cognitive impairment. This preliminary finding is derived from persistent cognitive difficulties related to chemotherapy regimens in a cohort of women with stage II and stage III breast cancer during the trajectory of treatment. Since the FAC group demonstrated greater perceived cognitive decline compared to the CMF group, the subjective cognitive impairment found in this study offers clinically useful information for patient management and may help clinicians select chemotherapy regimens with more tolerable side effects. This study may contribute towards the insights into the neurocognitive effects of anthracycline-based chemotherapy.

Only IL-8 showed a significant correlation with subjective cognitive deterioration after completion of chemotherapy treatment. It is crucial to note that despite the other inflammatory cytokines not showing any significance, there was a steady increase in inflammatory markers during the course of the treatment. Although this was not scientifically significant, it is important to remember that inflammation can be mild in a therapeutic setting, which is why objective cognitive alterations are frequently overlooked and not seen in clinical practice [6].

This finding is clinically meaningful for the management of breast cancer patients in the clinical setting. This highlights the importance of incorporating neuropsychology into an oncology setting. This is an important consideration in order to identify individuals at risk and to augment oncology care with cognitive interventions to enhance brain reserves of patients that will undergo cancer treatment. For this purpose, a specific assessment toolkit needs to be designed and implemented in the clinical setting.

## Figures and Tables

**Table 1:** Demographic characteristics of participants.

Variable	CMF Regimen	FAC Regimen
**Mean age (in years)**	48.2	47.1
**Ethnic group**		
Black African	53	60
**Marital status**		
Married	36	31
Divorced	03	07
Separated	07	01
Widowed	07	08
Single	00	13
**Educational level**		
Primary	09	08
Middle school	25	28
High school	18	23
Tertiary	01	01
**Employment status**		
Employed (Full-time)	03	07
Employed (Part-time)	02	01
Unemployed	48	52
**Family history (BC)**	05	03
**BC stage at diagnosis**		
II	22	32
III	31	28

**Table 2:** FACT-Cog scores during period of chemotherapy treatment.

	CMF (n = 53)			FAC (n = 60)			*p* Value: CMF vs. FAC
	Mean (±SD) FACT-Cog Cognitive Scores	95% CI	*p* Value:	Mean (±SD) FACT-Cog Cognitive Scores	95% CI	*p* Value:	
T_0_ (Baseline)	51.2 (±6.75)	49.3–53.0		52.9 (±8.50)	50.7–55.1		
T_1_ (Cycle 3)	48.9 (±5.89)	47.3–50.5		48.4 (±7.83)	46.4–50.4		
T_2_ (Cycle 6)	47.0 (±8.79)	44.5–49.4		44.0 (±10.83)	41.2–46.8		
Decrease	2.3 (±7.15)	0.3–4.3	0.023	4.5 (±11.26)	1.6–7.4	0.003	0.229
T_0_ → T_1_			(df = 52, t = 2.34)			(df = 59, t = 3.08)	(df = 111, t = 1.21)
Decrease	4.2 (±11.13)	1.2–7.3	0.008	8.9(±14.37)	5.2–12.6	<0.001	0.056
T_0_ → T_2_			(df = 52, t = 2.75)			(df = 59, t = 4.81)	(df = 111, t = 1.93)

Note: Cyclophosphamide, methotrexate, fluorouracil (CMF) and fluorouracil, adriamycin, cyclophosphamide (FAC). Significance level set as *p* < 0.05.

**Table 3:** Pearson’s correlation between levels of inflammatory cytokines and subjective cognitive function from baseline (T_0_) to completion of chemotherapy (T_2_).

Cytokines	FACT-Cog T_0_		FACT-Cog T_1_		FACT-Cog T_2_	
	r	*p*	r	*p*	r	*p*
Baseline before chemo						
IL-1β T0	0.019	0.838				
IL-6 T0	0.042	0.662				
IL-8 T0	−0.050	0.598				
TNF-α T0	−0.106	0.264				
Midway through chemo						
IL-1β T_1_			0.205	0.030		
IL-6 T_1_			−0.043	0.648		
IL-8 T_1_			0.103	0.280		
TNF-α T_1_			−0.191	0.042		
Completion of chemo						
IL-1β T_2_					0.020	0.831
IL-6 T_2_					−0.139	0.141
IL-8 T_2_					−0.185	0.050
TNF-α T_2_					−0.170	0.071

Note: Functional Assessment of Cancer Cognition (FACT-Cog), interleukin-1 beta (IL-1β), interleukin-6 (IL-6), interleukin-8 (IL-8), and tumour necrosis factor-alpha (TNF-α). Significance level set as *p* < 0.05.

## Data Availability

The data presented in this study are available on reasonable request from the corresponding author. The data are not publicly available due to ethical requirements.
